# Effects of Zinc Oxide and Silicon Dioxide Nanoparticles on Physiological, Yield, and Water Use Efficiency Traits of Potato Grown under Water Deficit

**DOI:** 10.3390/plants12010218

**Published:** 2023-01-03

**Authors:** Wadei A. Al-Selwey, Abdullah A. Alsadon, Abdullah A. Ibrahim, Joselito P. Labis, Mahmoud F. Seleiman

**Affiliations:** 1 Plant Production, College of Food and Agriculture Sciences, King Saud University, Riyadh 11451, Saudi Arabia; 2 King Abdullah Institute for Nanotechnology, King Saud University, Riyadh 11451, Saudi Arabia; 3 Department of Crop Sciences, Faculty of Agriculture, Menoufia University, Shibin El-kom 32514, Egypt

**Keywords:** nanoparticles, water deficit, zinc oxide NPs, silicon dioxide NPs, photosynthesis, WUE, potato

## Abstract

Water deficit is a major challenge for sustainable global food security, especially, in arid and semi-arid regions. Nanotechnology is regarded as an effective tool for managing a wide range of environmental stresses by providing novel and practical solutions. A field experiment was conducted to assess the effects of zinc oxide nanoparticles ‘ZnO NPs’ (0, 50, 100 ppm) and silicon dioxide nanoparticles ‘SiO_2_ NPs’ (0, 25, 50 ppm) as an exogenous application on the physiological indices, total yield and water use efficiency (WUE) of potato under water deficit conditions (50%, 75%, and 100% of crop evapotranspiration (ETc) water requirements). Water deficit significantly decreased most physiological indices and yield traits of potato, but increased proline content and WUE. In contrast, exogenous application of ZnO NPs and SiO_2_ NPs to plants grown under different water deficit treatments resulted in an increase in leaf gas exchange, leaves relative water contents (LRWC), photosynthetic pigments, and leaf green index. Under different water deficit treatments, the highest total yield and harvest index traits were obtained from plants treated with ZnO-NPs-100 ppm followed by 50 ppm of ZnO and SiO_2_ NPs, respectively. The highest WUE was recorded when the potato plants were irrigated with 50% ETc and exogenous treated with 100 ppm of ZnO NPs compared with fully irrigated plants. In conclusion, the exogenous application of ZnO NPs (100 ppm) can significantly mitigate the water deficit stress and improve the physiological, yield, and WUE of potato grown in arid regions under water deficit conditions.

## 1. Introduction

Potato (*Solanum tuberosum* L.) is one of the important food crops for human populations and is an important source of nutrients [1]. Worldwide, it ranks fourth as a strategic and economic crop following *Triticum aestivum* L., *Oryza sativa* L., and *Zea mays* L. [2]. Food security is of great importance due to the increase in world population, which is expected to be 10 billion by 2050 [3]. Climate change coupled with the diminishing cultivable agricultural land and freshwater resources has generated high demand for new technologies and processes to increase agricultural production [4]. Therefore, crop production should be significantly increased to meet the ever-increasing demand for food worldwide [5]. Abiotic stresses imposed by harsh environmental conditions severely affect crop yield and quality traits. The major abiotic stresses are drought [6], salinity [7], and temperature [8]; which cause a drastic yield reduction in most crops. 

Potato crop is sensitive to soil water deficits since all potato growth stages are affected by water shortages. Tuber initiation and bulking are considered the most sensitive stages that are associated with tuber yields and quality [9,10]. Water deficit conditions can decrease photosynthesis rate, stomatal conductance, transpiration rate, and leaf green index. During the water deficit conditions, chlorophyll content can be significantly affected while a high level of proline can be accumulated in the leaves [11,12]. In arid regions where water shortage and droughts prevail, water scarcity is considered the main concern in the agricultural system [13]. These challenges have forced farmers to use low-quality water, and thus drought tolerance crops and suitable strategies are quite important for such environmental conditions. Maximizing water use efficiency (WUE) may be a more beneficial option for farmers than maximizing crop yield [14]. 

Nanoparticles (NPs) include organic or inorganic materials with sizes ranging from 1 to 100 nm, and they have been commonly used worldwide in recent years [4,15]. NP fertilizers increased crop productivity and reduced production costs [16,17]. Various kinds of nanomaterials have shown promise in promoting sustainable agriculture as they help to improve agricultural production by increasing the efficiency of inputs and minimizing yield losses [18]. Thus, the application of NPs is gaining momentum in modern agriculture via exhibiting promising roles in enhanced crop productivity, maintaining soil health, precision farming, and crop improvement [19]. Drought tolerance is a complex character of high importance for the potato crop. Therefore, nanotechnology techniques can provide clear guidelines for enhancing potato drought tolerance and yield sustainability. Studies on several crops such as wheat [20], tomato [21], eggplant [22], and cucumber [23], have shown that NPs have a positive impact on plants’ response to water shortage conditions. Therefore, this present study aimed to investigate the effects of nanoparticles (zinc oxide ‘ZnO-NPs’ and silicon dioxide ‘SiO_2_-NPs’) as exogenous application and their role in physiological, yield, and water use efficiency (WUE) of potato plants grown under water deficit stress in arid regions. Our hypothesis was that the application of ZnO-NPs or SiO_2_-NPs could enhance physiological and improve yield and water use efficiency (WUE) of potato grown under water deficit stress.

## 2. Results and Discussion

### 2.1. Characterization of ZnO and SiO_2_ NPs

**Figure 1:**
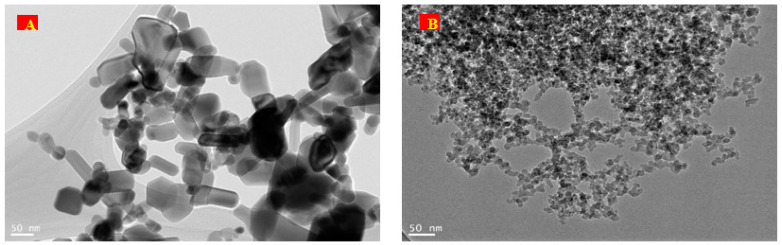
The micrographs and structure of ZnO (**A**) and SiO_2_ (**B**) NPs using TEM.

The micrographs of TEM for ZnO and SiO_2_ NPs are shown in Figure 1A,B, respectively. The surface image of the ZnO NPs showed a smooth semispherical to hexagonal wurtzite shape and few were non-spherical monoclinic particles, while the surface image of the SiO_2_ NPs showed a non-smooth and nearly spherical shape with a small size. 

**Figure 2:**
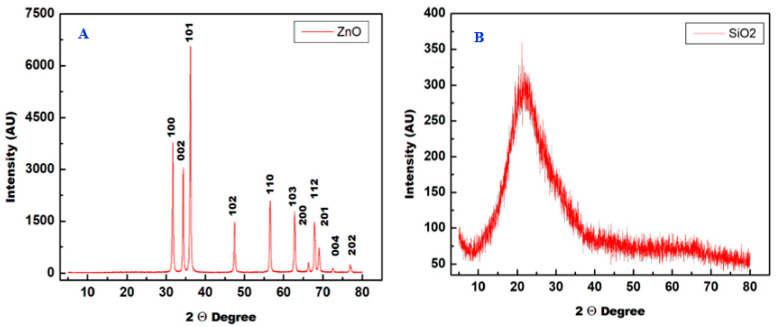
X-ray diffraction (XRD) pattern recorded for ZnO NPs (**A**) and SiO_2_ NPs (**B**).

The XRD of ZnO and SiO_2_ NPs were recorded on MiniFlex-600 (Rigaku) X-ray diffractometer using Cu Kα radiation (λ = 1.54 Å) at 40 kV (Figure 2A,B). The XRD spectrum of pure ZnO NPs is displayed in Figure 2A. Sharp and narrow diffraction peaks indicated the size and crystallinity of the ZnO NPs. These sharp and narrow peaks appeared at 2θ = 31.76°, 34.42°, 36.25°, 47.53°, 56.59°, 62.85°, 66.37°, 67.94°, 69.08°, 72.56°, and 77.03° at a reflecting plane (100), (002), (101), (102), (110), (103), (200), (112), (201), (004), and (202), respectively (Figure 2A). The sharp peaks indicate that the ZnO NPs were highly crystalline in nature.

The phase structure and purity of the SiO_2_ NPs were investigated by XRD, as shown in Figure 2B. The XRD pattern displayed a typical broad peak at 22°, which corresponded to the amorphous phase of the SiO_2_ NPs. This broad XRD reflection peak could be due to the small size of SiO_2_ NPs and incomplete inner structure and a high percentage of SiO_2_ NPs are amorphous [24,25]. Moreover, the absence of any other peaks indicates that the SiO_2_ NPs is free of any impurities.

### 2.2. Structure of Potato Leaf Stomata

**Figure 3:**
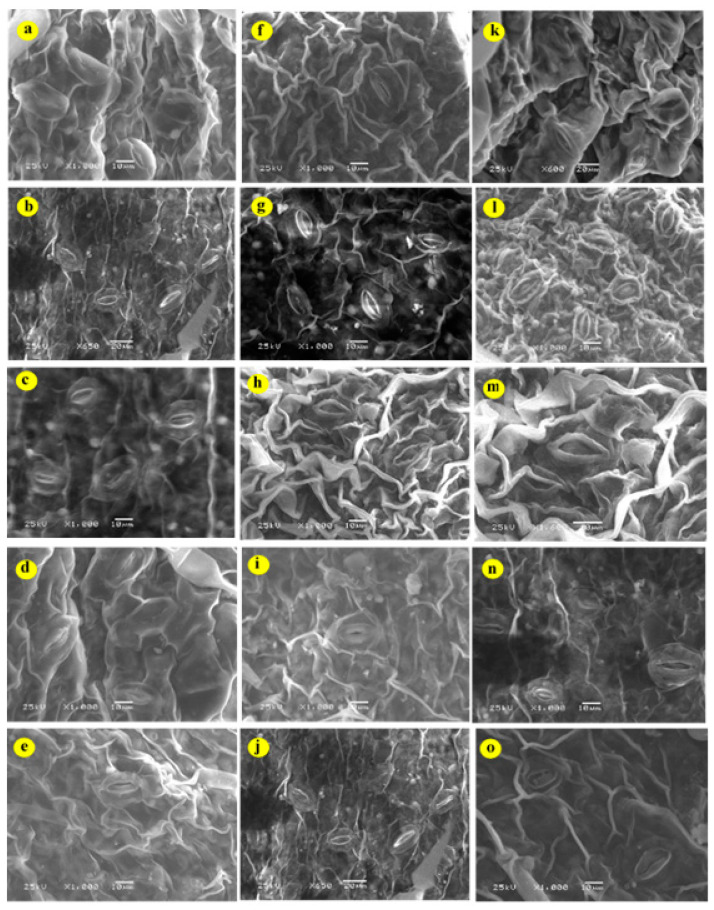
SEM images of the potato leaves and their stomata structure under water deficit (ETc%) and exogenous nanoparticles (NPs) treatments. (**a**–**e**) 50% ETc; (**a**) control; (**b**) ZnO-NPs-50 ppm; (**c**) ZnO-NPs-100 ppm; (**d**) SiO_2_-NPs-25 ppm, (**e**) SiO_2_-NPs-50 ppm; (**f**–**j**) 75% ETc; (**f**) control; (**g**) ZnO-NPs-50 ppm; (**h**) ZnO-NPs-100 ppm; (**i**) SiO_2_-NPs-25 ppm; (**j**) SiO_2_-NPs-50 ppm and (**k**–**o**) 100% ETc; (**k**) control; (**l**) ZnO-NPs-50 ppm; (**m**) ZnO-NPs-100 ppm; (**n**) SiO_2_-NPs-25 ppm; (**o**) SiO_2_-NPs-50 ppm.

As shown in the SEM image (Figure 3a,f), potato leaf stomata are closed under water deficit. The exogenous application of 100 (Figure 3c,h,m) or 50 ppm ZnO NPs (Figure 3b,g,l) and 50 ppm SiO_2_ (Figure 3e,j,o) maintained the stomatal structure and prevented its deformation in comparison to the untreated plants with NPs when grown under water deficit. However, the treatment of ZnO NPs or SiO_2_ NPs considerably enhanced the performance of photosynthesis, stomatal conductance, and intercellular CO_2_ concentration (Table 1), especially in plants subjected to 100 and 50 ppm of ZnO-NPs. Stomatal closure is considered an important factor that can lead to a reduction in the gaseous exchange in plants grown under abiotic stress such as salt stress [26] and drought stress [27]. Nano-particles can enhance photosynthesis and related attributes by hastening the splitting of water, and the electron exchange via redox reactions [28]. In addition, NPs have a positive potential to mitigate the negative impacts of water deficit on plants [4] by improving soil water-holding capacity. 

### 2.3. Effects of ZnO and SiO_2_ NPs on Leaf Gas Exchange, LRWC and Proline Content of Potato Grown under Different Water Deficit Treatments

Water deficit negatively affected leaf gas exchange and LRWC traits, in particular when plants were subjected to 50% ETc in comparison to 100% ETc and/or 75% ETc (Table 1). Concerning proline content, the highest water deficit stress (i.e., 50% of ETc) resulted in the highest proline content in potato plant leaves. These results confirmed the findings of Alhoshan et al. [11] and Mahmud et al. [12] who stated that a significant increase in the proline content occurred in plants grown under water deficit. However, the application of NPs treatments such as ZnO-NPs (i.e., 50, 100 ppm) and SiO_2_-NPs (i.e., 25, 50 ppm) improved leaf gas exchange traits, LRWC, and proline content of potato plants when subjected to water deficit (Table 1). For instance, the application of ZnO-NPs at the rate of 100 ppm resulted in the highest leaf gas exchange traits and LRWC, and the lowest proline content, followed by the rate of 50 ppm from ZnO and SiO_2_ NPs, respectively, compared to untreated plants (Table 1). This can be due to the role of ZnO and SiO_2_ NPs that can enhance the rate of photosynthesis by improving gas exchange, chlorophyll fluorescence, carbonic anhydrase activity, and enhanced proline and relative water contents [19].

**Figure 4:**
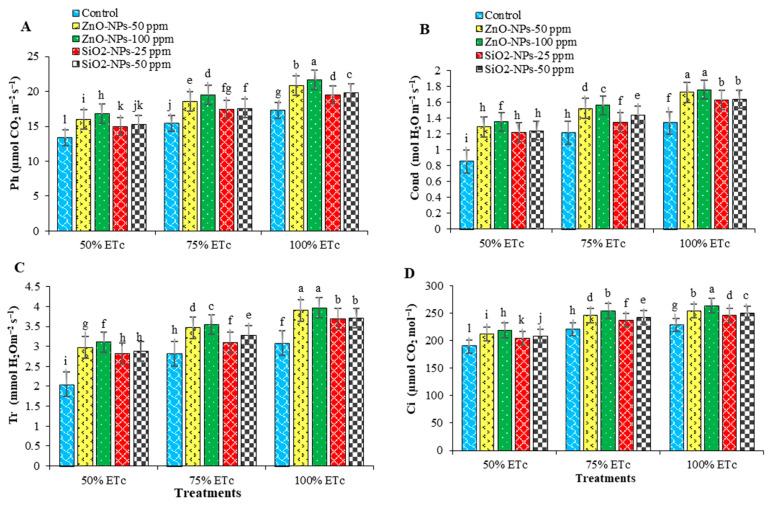
Interaction effects of water deficit (ETc) and exogenous nanoparticles (NPs) treatments on leaf gas exchange of potato; photosynthesis rate (**A**), stomatal conductance (**B**), transpiration rate (**C**) and intercellular CO_2_ concentration (**D**). Columns with different letters showed significant differences according to LSD at *p* ≤ 0.05. Bars = standard error of means (SEM).

The application of different exogenous NPs minimized the negative impact of water deficit. Under the water deficit treatments, ZnO-NPs-100 ppm gave the highest significant mean values of leaf gas exchange traits, followed by 50 ppm of ZnO and SiO_2_ NPs, respectively (Figure 4). Photosynthesis rate, stomatal conductance, transpiration rate, and intercellular concentration of CO_2_ traits are vulnerable to adverse environmental conditions such as drought, salinity, and heat [29]. Therefore, drought causes damage to photosynthetic pigments and thylakoid membranes [30,31]. ZnO and SiO_2_ NPs enhanced net photosynthetic rate, transpiration rate, and stomatal conductance [32,33].

**Figure 5:**
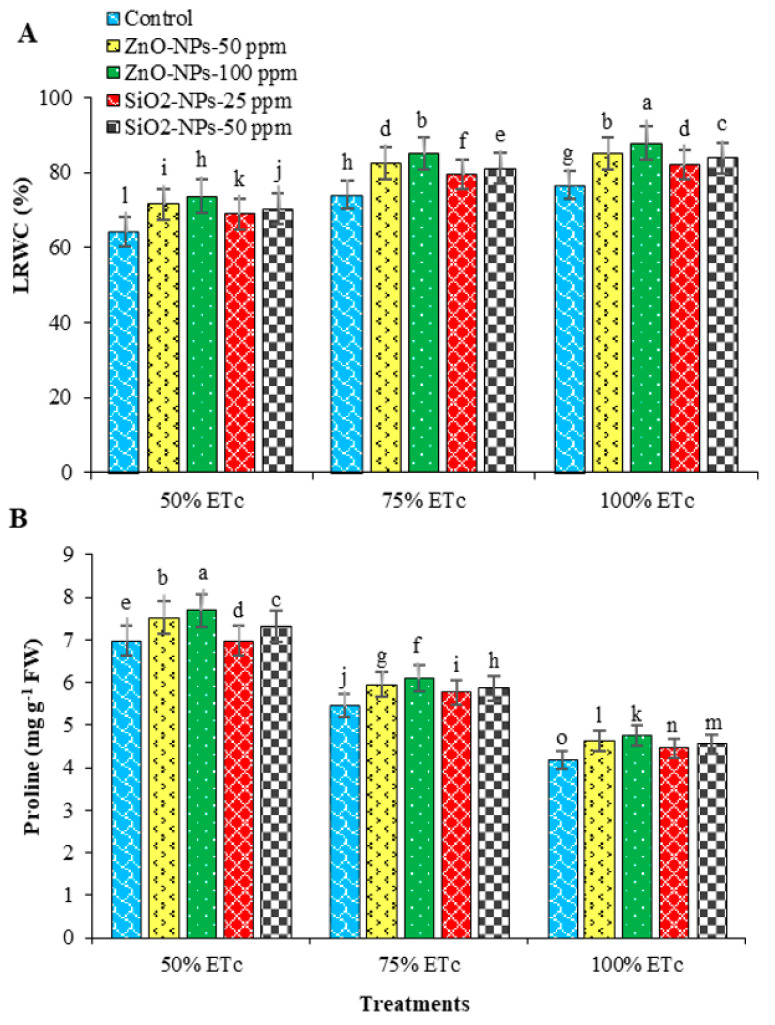
Interaction effects of water deficit (ETc) and exogenous nanoparticles (NPs) treatments on the relative water content of leaves (**A**) and proline content (**B**) of potato. Columns with different letters showed significant differences according to LSD at *p* ≤ 0.05. Bars = standard error of means (SEM).

The results presented in Figure 5A show that the highest LRWC was obtained from plants treated with ZnO-NPs-100 ppm followed by 50 ppm of ZnO and SiO_2_ NPs, respectively as compared with plants were irrigated with 100% and 75% of ETc. In contrast, the lowest values of LRWC were recorded from untreated plants and grown under the highest water deficit treatment (50% ETc). The application of ZnO NPs mitigated the negative effects of water deficit in terms of improving LRWC and related traits. This can be associated with the improvement of leaf anatomical structures which can enhance the photosynthetic efficiency in water-stressed plants [22]. SiO_2_ NPs at a rate of 50 ppm enhanced the leaves RWC of green pea grown under water deficit [34]. On the other hand, proline content in potato leaves was the highest under the irrigation treatment at 50% of ETc and treated with ZnO-NPs-100 ppm and 50 ppm of ZnO and SiO_2_ NPs, respectively. On the contrary, the lowest proline levels were recorded in untreated plants with NPs and grown under the irrigation treatment of 100% ETc (Figure 5B). According to Marco et al. [35] and Aghaie et al. [36], proline accumulation in plants grown under abiotic stress can be caused by either induction of expression of proline biosynthesis genes or repression of its degradation pathway genes. Furthermore, the content of proline increased under water stress conditions when ZnO NPs were applied to cucumber seedlings.

### 2.4. Effects of ZnO and SiO_2_ NPs on Chlorophyll-a, Chlorophyll-b, Total Chlorophyll, Carotenoids and Leaf Green Index of Potato Grown under Different Water Deficit Treatments

Water deficit reduced the chlorophyll-a, chlorophyll-b, total chlorophyll, carotenoids, and leaf green index traits as compared with a plant well-watered, particularly at high water deficit 50% of ETc (Table 2). This response might be due to ROS destructive effects on chloroplast [37]. The interaction between water deficit and exogenous NPs treatments was highly significant in chlorophyll-a, chlorophyll-b, total chlorophyll, and carotenoids, while no significant differences in leaf green index traits. Exogenous application of ZnO and SiO_2_ NPs, specifically 100 ppm ZnO NPs, reduced this damage under drought conditions. The enhancement of potato growth in the current study as a result of ZnO NPs applications can be due to the positive role of ZnO NPs in chloroplast development [38]. A plentiful supply of Zn promotes plant growth by improving photosynthesis and enzymatic activity [39]. There is evidence in the literature that ZnO NPs modulate the expression of microRNAs, which play an important role in the formation and activation of numerous mechanisms in plants under a variety of stressful conditions [40]. Previous studies have also shown that ZnO NPs enhance chlorophyll and photosynthesis activity in drought-stressed plants [20,22]. Furthermore, ZnO NP treatment increased Rubisco enzyme activity, which was directly related to increased photosynthetic activity [41].

### 2.5. Effects of ZnO and SiO_2_ NPs on Total Yield, Harvest Index and WUE of Potato Grown under Different Water Deficit Treatments

Potato total yield, harvest index, and WUE varied with the application of different water deficit and exogenous NPs treatments (Table 3). The lowest total yield and harvest index were obtained from the lowest level of irrigation (50% ETc). This result indicates that a high irrigation level increased the total yield. On the other hand, exogenous ZnO and SiO_2_ NPs treatments increased total yield and harvest index as compared with control treatment (non-NPs). For instance, the exogenous application of ZnO-NPs-100 ppm and 50 ppm of ZnO-NPs and SiO_2_ increased the total yield and harvest index. However, the highest mean values of total yield and harvest index were obtained using the treatments at 100% followed by 75% ETc with ZnO-NPs-100 ppm, respectively. The highest yield was found when 100 ppm of ZnO NP was applied to fully or deficit-irrigated plants [22]. These findings support previous research of Etienne et al. [42] who reported that plants require micronutrients in addition to macronutrients for optimal development and yield potential. The significantly highest mean values of WUE resulted from 50% ETc treatment, followed by 75% ETc treatment (Table 3). On the contrary, the lowest WUE was observed by the application of 100% ETc treatment. A similar tendency was observed by Aziz et al. [43] who reported that higher WUE for the treatment of 50% ETc as compared to 100 or 75% ETc. In the same trend, El-Sawy et al. [44] and Nagaz et al. [45] found that WUE increased with decreased irrigation water levels. The highest value of this trait was recorded under water stress at 40 or 50% ETc. The interaction between water deficit and exogenous NPs treatments was highly significant in WUE. The use of NPs in agriculture will decrease the abiotic stress caused by drought and increase water use efficiency in plants [46].

## 3. Materials and Methods

### 3.1. Characterization of ZnO and SiO_2_ Nanoparticles

The ZnO and SiO_2_ NPs (Sigma-Aldrich, Saint-Louis, MO, USA) were characterized at King Abdullah Institute for Nanotechnology, King Saud University, Riyadh, Saudi Arabia. X-ray powder diffraction (XRD) measurements in MiniFlex-600 (Rigaku) with Cu Kα radiation (λ = 1.5406 Å) at a voltage of 40 kV and current of 15 mA with 2θ ranging from 10°–80° were used for the analysis of crystalline purity and phases of materials. The crystallographic data for materials were analyzed using the Fullprof software [47]. Crystallite size was obtained by Equation (1) according to Shaltout and Abdelkader [48],

(1) $$D=\frac{0.94\times \lambda }{\beta \times cos\;\theta }$$

where D is the crystallite size, 0.94 is the factor which depends on the particles shape, λ is the Cu Kα radiations (λ = 1.54 Å), β is full width at half maximum (FWHM) of the selected diffraction peak corresponding to 101 plane and θ is the Bragg angle obtained from 2 θ value corresponding to maximum intensity peak in XRD pattern. The values of XRD were subjected to Fullprof software and compared with PDF No. 01-080-0075 and 29-0085 for ZnO and SiO_2_ NPs respectively. The surface morphology and composition of NPs were characterized by a transmission electron microscope (TEM) (JEM-1010, Tokyo, Japan).

### 3.2. Experimental Site

**Figure 6:**
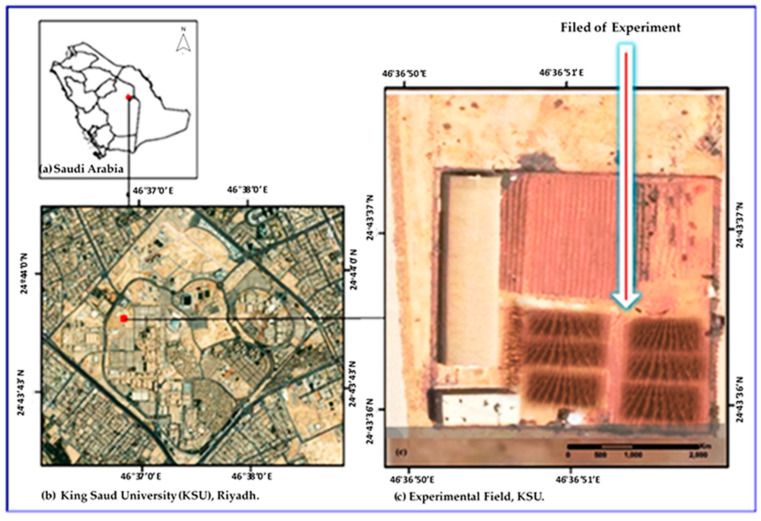
The location of the field experiment.

This study was carried out under field conditions at Plant Production Research Unit, College of Food and Agriculture Sciences, King Saud University, Riyadh, Saudi Arabia (Figure 6). Weather conditions were recorded from the on-site weather station (Table 4).

Soil samples were collected from the experimental field prior to the beginning of the experiment and the physical, and chemical properties of the experimental soil are presented in Table 5, while water chemical analysis is presented in Table 6.

### 3.3. Plant Materials, Experimental Layout, and Treatments

Certified potato seed tubers (*Solanum tuberosum* L. cv. Hermes) were obtained from the Saudi Agricultural Development Company, Riyadh. This cultivar is considered a medium-maturing plant and is suitable for industry [49]. Potato tubers were cultivated on 25th September 2021. The average tuber weight was 59–64 g, with a diameter ranging between 45–55 mm. Potato tubers were planted in rows, 100 cm apart with 40 cm between plants in each row. A split plot in a randomized complete block design with three replicates was used. Irrigation treatments (irrigation at 50% crop evapotranspiration (ETc); 75% ETc and 100% ETc) were assigned to the main plots, while zinc oxide (ZnO-NPs, 50 and 100 ppm) and silicon dioxide (SiO_2_-NPs 25 and 50 ppm) were placed in the sub-plots. Drip irrigation was used and the amount of irrigation water was estimated using Equation (2):

(2) $$\mathrm{ETc}={\mathrm{ET}}_{o}\times \mathrm{Kc}$$

where ETc = crop evapotranspiration (mm day^−1^), Kc = crop coefficient and ET_o_ = reference evapotranspiration (mm day^−1^). 

FAO CROPWAT software ver. 8 was used for the estimation of ET_o_. This software uses the modified FAO Penman–Monteith equation to estimate the ET_o_, as reported by Allen et al. [50]. The daily climatic data were collected from the on-site meteorological station (Table 1) and were applied to the modified FAO Penman–Monteith equation as shown in Equation (3):

(3) $${\mathrm{ET}}_{o}=\frac{0.408\Delta \;\left(R_{n}-G\right)+\gamma \frac{900}{T_{mean}+273}\;u_{2}\;\left(e_{s}-e_{a}\right)\;\;}{\Delta +\gamma \left(1+0.34\;u_{2}\right)}$$

where:

ET_o_ = reference evapotranspiration (mm day^−1^).
*R_n_* = net radiation at the crop surface (MJ m^−2^ day^−1^).
*G* = soil heat flux density (MJ m^−2^ day^−1^).
*T*_mean_ = mean daily air temperature at 2 m height (°C).
*u*_2_ = wind speed measured at 2 m height (m s^−1^).
*e_s_* = saturation vapour pressure (kPa).
*e_a_* = actual vapour pressure (kPa),
*e_s_* − *e_a_* = the saturation vapour pressure deficit (kPa).
Δ = the slope vapour pressure curve (kPa °C^−1^).
*γ* = the psychrometric constant (kPa °C^−1^).

**Figure 7:**
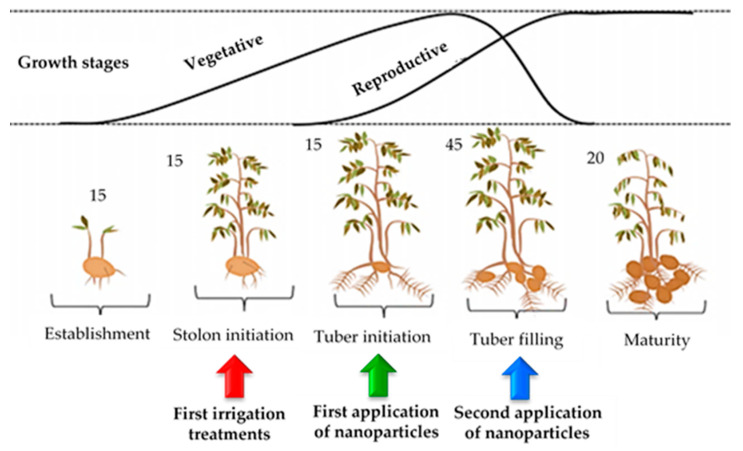
Schematic illustration of the potato growth stages. Exogenous spray application with zinc oxide and silicon dioxide nanoparticle were conducted at two reproductive periods (i.e., 45 and 65 DAP) under water deficit treatments.

The total amount of consumptive water for the 0.50 ET, 0.75 ET, and 1.00 ET treatments were 2946, 4419, and 5892 m^3^ ha^−1^, respectively. The irrigation water volume of full irrigation was reduced to 75% and 50%. ZnO and SiO_2_ NPs in the concentration of 50, 100, and 25, 50 ppm, respectively were prepared with double distilled water. Tween 20 (0.05%) was added in solution as a surfactant to ensure uniform retention and coverage of the solution on the leaf surface. The irrigation treatments were applied at 35 days after planting (DAP). The applications of exogenous spry of nanoparticles were applied at 45 and 65 DAP (Figure 7) 

Fertilization was applied as commonly recommended in commercial potato production, with the same quantity for all treatments via the drip irrigation system. Other recommended agricultural practices of potato production, plant protection against diseases and insects, were performed as commonly used in the commercial production of potato [51]. 

### 3.4. Measurements

#### 3.4.1. Leaf Gas Exchange 

The photosynthesis rate, stomatal conductance, transpiration rate, and intercellular CO_2_ concentration were determined in the field on a sunny day at 10–12 a.m. using an LI-6400 photosynthesis system (Li-Cor, Inc., Lincoln, NE, USA). Third, completely expanded leaf (from the apex) was exposed to 1200 μmol (photon) m^–2^ s^−1^ PPFD, chamber temperature of 25 °C, CO_2_ concentration of 350 ± 10 μmol (CO_2_) mol^−1^, and RH of 50–55% for each measurement. 

#### 3.4.2. Relative Water Content of Leaves (LRWC) 

Leaf relative water content was determined based on fresh, turgid, and dry weights of leaf discs. After measuring fresh weight, they were placed into containers with distilled water for 24 h until constant weight. Turgid weight was calculated for each sample of leaves. Dry weight was obtained after drying leaves at 70 °C in the oven for 72 h till constant weight. LRWC percentage (LRWC, %) was determined using Equation (4) according to Kafi et al. [52]

(4) $$\mathrm{LRWC}\;\left(\%\right)=\frac{\mathrm{FW}-\mathrm{DW}}{\mathrm{TW}-\mathrm{DW}}\times 100$$

where FW is the leaf fresh weight, TW is the turgid weight, and DW is the leaf’s dry weight.

#### 3.4.3. Microscopic Observations of Leaf Stomata

Leaf samples were randomly collected and cut into about (1 cm) in the middle of the lamina and then put in a test tube containing glutaraldehyde. Small pieces (approximately 0.5 × 0.5 cm) were taken from the areas between the margin and midrib of fresh leaves and directly fixed in 2.5% glutaraldehyde in a 0.2 M of phosphate buffer stock solution (pH 7.2) at 4 °C for 24 h followed by two rinses in the same buffer for 15 min and post-fixation in osmium tetroxide (OsO_4_) for 1 h. The tissue pieces were washed three times in a sodium cacodylate solution for 30 min. The samples were dehydrated in a series of ascending graded ethanol (25%, 50%, 75%, 90%, and 100%) for 10 min in each solution ratio. Then, the specimens were transferred to a vacuum chamber connected to a rotary pump. Dry specimens were removed from the vacuum chamber and stored in a desiccator. The dried samples were mounted in metal stubs and sputter coated with a thin conductive film of gold. The coated samples were examined and photographed using scanning electron microscopy (SEM) with a high resolution of 3.0 nm (JEOL Ltd., Tokyo, Japan) at 20 kV [53].

#### 3.4.4. Proline Content 

Proline content of leaves was measured following the method of Claussen [54]. Extraction procedure and colorimetric determination with acidic ninhydrin reagent were prepared by warming 3.75 g ninhydrin in 90 mL glacial acetic acid and 60 mL of 6 molar phosphoric acid, with agitation, until dissolved. Proline was extracted by grinding (0.25 g) samples of wet plant (leaves) in a ceramic mortar with 5 mL of 3% (*w*/*v*) aqueous sulfosalicylic acid and was transferred to a 2 mL tube and debris was removed by centrifuging at 4000 rpm for 10 min to pellet the sample tissue. Then, 2 mL of supernatant was transferred to a tube and reacted with an equal volume of each glacial acetic acid and ninhydrin reagent and incubated for 1 h at 100 °C. The reaction was terminated by placing the reaction tubes in an ice bath for 2 min. The reaction mixtures were vigorously mixed with 4 mL toluene (C_6_H_5_-CH_3_) for 15–20 s. After warming at 25 °C, a standard curve was calculated by measuring dilutions of proline stock solution. proline was measured at a wavelength of 520 nm using a spectrophotometer (T80 UV-Visible Spectrophotometer, PG instruments, Lutterworth, UK).

#### 3.4.5. Photosynthetic Pigments Content 

The chlorophyll a (Chl- a), chlorophyll b (Chl- b), total chlorophyll, and carotenoids of leaves were spectrophotometrically measured (T 80 UV/Visible Spectrophotometer, PG Instruments Ltd., Lutterworth, UK) according to Moran and Porath [55] and Wellburn [56]. Photosynthetic pigment content of plants was extracted by ground a 0.5 g fresh weight of leaves in 10 mL 80% aqueous acetone for 5 min. The extract was centrifuged at 15,000× *g* for 5 min. The supernatant was then taken and diluted to 25 mL by 80% aqueous acetone to a suitable concentration for spectrophotometric measurements. The absorbance was measured against a blank of pure 80% aqueous acetone at three wavelengths of 663, 645, and 480 nm by using a glass cell whose optical path thickness is 1 cm, respectively. The contained chlorophyll a (Equation (5)), chlorophyll b (Equation (6)), total chlorophyll a + b (Equation (7)), and carotenoids (mg/g fresh weight) (Equation (8)) were determined according to Arnon [57].

(5) Chlorophyll (a)=[(12.7×O. D 663)−(2.69×O. D 645)]×V/1000×W

(6) Chlorophyll (b)=[(22.9×O.D 645)−(4.68×O.D 663)]×V/1000×W

(7) Total chlorophyll=[(20.2×O.D 645+(8.02× O.D 663)]×V/1000 W

(8) Carotenoids (Car)=[O.D 480+(0.114×O.D 663)]−(0.638×O.D 645)

where O.D: optical density of the extract at the wavelength shown, V: volume of extract (ml), and W: weight of the fresh leaves (g).

#### 3.4.6. Leaf Green Index (SPAD Reading) 

Three leaflets of the third fully developed leaf (from the apex) were taken to determine the leaf green index in the field at 11–12 a.m. using a SPAD-502 m (Konica Minolta, Tokyo, Japan). The data were recorded in triplicate from each leaf.

#### 3.4.7. Total Yield and Harvest Index

The total harvested tubers from each plot were weighted and then calculated as tons per hectare. Harvest index (HI) was calculated as: total tubers yield/total biomass.

#### 3.4.8. Water Use Efficiency 

The water use efficiency (WUE) was determined using Equation (9) according to Reddy and Reddi [58]:

(9) $$\;\mathrm{WUE}\;\left({\mathrm{kg}\;m}^{-3}\right)=\frac{\mathrm{Total}\;\mathrm{tubers}\;\mathrm{yield}\;\left({\mathrm{kg}\;\mathrm{ha}}^{-1}\right)}{\mathrm{Water}\;\mathrm{applied}\;\left(m^{3}{\;\mathrm{ha}}^{-1}\right)}$$

#### 3.4.9. Statistical Analysis

All collected data from the effects of zinc oxide and silicon dioxide nanoparticles as exogenous application on physiological, yield, and WUE traits of potato plants grown under water deficit stress were arranged and statistically analyzed through ANOVA using the statistical analysis program (SAS GLM procedure version 9.2, SAS Institute Ltd., Cary, NC, USA). The differences among the different means of treatments were tested using the LSD test at a probability *p* ≤ 0.05.

## 4. Conclusions

The exogenous application of ZnO and SiO_2_ NPs significantly mitigated water deficit stress (i.e., 50 and 75% ETc) and enhanced photosynthesis and WUE as well as improved the productivity of potato. The application of ZnO NPs at 100 ppm significantly surpassed all other treatments either 50 ppm ZnO NPs or SiO_2_ NPs (25 and 50 ppm) in terms of mitigating the water deficit stress and improving the physiological, WUE, and yield of potato.

## Figures and Tables

**Table 1:** Effects of water deficit and exogenous nanoparticles (NPs) treatments on leaf gas exchange traits, relative water content of leaves (LRWC), and proline content of potato.

	Traits	Photosynthesis Rate (µmol CO_2_ m^−2^ s^−1^)	Conductivity (mol H_2_O m^−2^s^−1^)	Transpiration Rate (mmol H_2_O m^−2^s^−1^)	Intercellular CO_2_ (Ci) (µmol CO_2_ mol^−1^)	LRWC (%)	Proline (mg/g^−1^ FW)
Treatments							
Irrigation levels (ETc)							
50% ETc		15.27 c	1.19 c	2.77 c	206.37 c	69.72 c	7.38 a
75% ETc		17.69 b	1.41 b	3.24 b	239.67 b	80.41 b	5.82 b
100% ETc		19.79 a	1.62 a	3.67 a	248.10 a	83.09 a	4.52 c
LSD _0.05_		0.09	0.02	0.05	0.82	0.17	1.51
Nano treatment (NPs)							
Control		15.33 e	1.13 e	2.65 e	212.37 e	71.64 e	5.53 e
ZnO-NPs-50 ppm		18.48 b	1.51 b	3.45 b	237.17 b	79.67 b	6.03 b
ZnO-NPs-100 ppm		19.29 a	1.56 a	3.54 a	245.45 a	82.17 a	6.18 a
SiO_2_-NPs-25 ppm		17.28 d	1.40 d	3.21 d	228.63 d	76.80 d	5.73 d
SiO_2_-NPs-50 ppm		17.53 c	1.44 c	3.29 c	233.28 c	78.42 c	5.91 c
LSD _0.05_		0.15	0.02	0.04	0.97	0.36	1.56

Values with different letters in each column are significantly differed at *p* ≤ 0.05. LSD = least significant difference.

**Table 2:** Effects of water deficit and exogenous nanoparticles (NPs) treatments on chlorophyll-a, chlorophyll-b, total chlorophyll, carotenoids, and leaf green index traits of potato.

Irrigation Levels	Nano Treatment (NPs)	Chlorophyll a (mg/g^−1^ FW)	Chlorophyll b (mg/g^−1^ FW)	Total Chlorophyll (mg/g^−1^ FW)	Carotenoids (mg/g^−1^ FW)	Leaf Green Index (SPAD)
50% ETc	Control	1.35 k	0.48 j	1.83 j	3.05 k	42.05 l
	ZnO-NPs-50 ppm	1.84 h	0.65 g	2.49 g	3.94 h	41.20 j
	ZnO-NPs-100 ppm	1.93 g	0.69 f	2.62 f	4.10 g	44.06 h
	SiO_2_-NPs-25 ppm	1.55 j	0.55 i	2.10 i	3.40 j	42.75 k
	SiO_2_-NPs-50 ppm	1.74 i	0.61 h	2.36 h	3.76 l	38.39 j
50% ETc mean		1.68 C	0.60 C	2.28 C	3.65 C	41.69 C
75% ETc	Control	1.85 h	0.66 fg	2.51 g	3.96 h	44.28 h
	ZnO-NPs-50 ppm	2.54 d	0.92 c	3.46 c	5.24 d	49.31 d
	ZnO-NPs-100 ppm	2.63 bc	0.96 b	3.59 b	5.40 be	50.82 b
	SiO_2_-NPs-25 ppm	2.16 f	0.78 e	2.94 e	4.53 f	47.52 f
	SiO_2_-NPs-50 ppm	2.23 e	0.82 d	3.05 d	4.66 e	48.50 e
75% ETc mean		2.28 B	0.83 B	3.11 B	4.76 B	48.09 B
100% ETc	Control	2.20 e	0.80 d	3.01 d	4.62 e	50.22 g
	ZnO-NPs-50 ppm	2.66 b	0.97 b	3.63 b	5.46 b	49.20 b
	ZnO-NPs-100 ppm	2.76 a	1.01 a	3.76 a	5.64 a	52.62 a
	SiO_2_-NPs-25 ppm	2.62 c	0.96 b	3.58 b	5.39 c	51.06 d
	SiO_2_-NPs-50 ppm	2.64 bc	0.96 b	3.60 b	5.42 bc	45.85 c
100% ETc mean		2.58 A	0.94 A	3.52 A	5.31 A	49.79 A
Nano treatments (NPs) means						
	Control	1.80 e	0.65 e	2.45 e	3.87 e	42.84 e
	ZnO-NPs-50 ppm	2.35 b	0.84 b	3.19 b	4.88 b	47.71 b
	ZnO-NPs-100 ppm	2.44 a	0.88 a	3.32 a	5.05 a	49.16 a
	SiO_2_-NPs-25 ppm	2.11 d	0.76 d	2.87 d	4.44 d	45.97 d
	SiO_2_-NPs-50 ppm	2.20 c	0.80 c	3.00 c	4.61 c	46.92 c
LSD _0.05_						
Irrigation levels (ETc)		0.01	0.02	0.02	0.02	0.03
Nano treatments (NPs)		0.02	0.01	0.03	0.04	0.25
ETc × NPs		0.04	0.02	0.06	0.07	NS

Values with different letters in each column are significantly differed at *p* ≤ 0.05. NS: not significant at *p* ≤ 0.05.

**Table 3:** Effects of water deficit and exogenous nanoparticles (NPs) treatments on total yield, harvest index, and water use efficiency of potato.

Irrigation Levels	Nano Treatments (NPs)	Total Yield (t ha^−1^)	Harvest Index (%)	WUE (kg m^−3^)
50% ETc	Control	22.519 m	32.431m	7.644 i
	ZnO-NPs-50 ppm	33.512 j	39.509 j	11.376 b
	ZnO-NPs-100 ppm	35.678 h	41.237 h	12.111 a
	SiO_2_-NPs-25 ppm	28.151 l	35.311 l	9.556 e
	SiO_2_-NPs-50 ppm	29.613 k	36.381 k	10.052 d
50% ETc mean		29.895 C	36.974 C	10.148 A
75% ETc	Control	27.595 l	37.632 j	6.245 l
	ZnO-NPs-50 ppm	39.624 e	45.784 e	8.967 f
	ZnO-NPs-100 ppm	46.152 b	52.699 b	10.444 c
	SiO_2_-NPs-25 ppm	34.511 i	40.892 h	7.810 h
	SiO_2_-NPs-50 ppm	36.331 g	42.330 g	8.221 g
75% ETc mean		36.843 B	43.867 B	8.337 B
100% ETc	Control	34.543 i	45.145 ef	5.863 m
	ZnO-NPs-50 ppm	45.323 c	51.474 c	7.692 hi
	ZnO-NPs-100 ppm	48.676 a	53.880 a	8.262 g
	SiO_2_-NPs-25 ppm	37.996 f	44.843 f	6.449 k
	SiO_2_-NPs-50 ppm	41.187 d	47.681 d	6.99 j
100% ETc mean		41.545 A	48.605 A	7.051C
	Nano treatments (NPs) means			
	Control	28.219 e	38.403 e	6.584 e
	ZnO-NPs-50 ppm	39.486 b	45.589 b	9.345 b
	ZnO-NPs-100 ppm	43.502 a	49.272 a	10.272 a
	SiO_2_-NPs-25 ppm	33.553 d	40.349 d	7.938 d
	SiO_2_-NPs-50 ppm	35.710 c	42.131 c	8.421 c
LSD _0.05_				
Irrigation levels (ETc)		0.072	0.164	0.051
Nano treatments (NPs)		0.347	0.455	0.079
ETc × NPs		0.600	0.787	0.137

Values with different letters in each column are significantly differed at *p* ≤ 0.05.

**Table 4:** Average of monthly weather conditions during growing period.

Months	Temperature (°C)		Relative Humidity (%)		Radiation Langley (day^−1^)	Wind Speed (ms^−1^)	Rainfall (mm)	Evaporation (mm)
	Max.	Min.	Max.	Min.				
September	39.67	25.27	30.77	9.67	399	3.22	0.00	10.45
October	36.35	21.45	41.81	13.97	340	2.48	0.00	9.67
November	29.17	15.83	52.93	22.47	267	2.44	0.00	5.74
December	23.00	11.39	61.35	27.81	225	2.75	0.15	3.57

**Table 5:** Soil physical and chemical properties.

Soil Texture				pH	EC (ds m^−1^)	Cations (mEq L^−1^)				Anions (mEq L^−1^)		
Clay%	Silt %	Sand %	Texture			K^+^	Na^+^	Mg^++^	Ca^++^	HCO_3_^−^	Cl^−^	SO_4_^−^
8.45	7.83	83.72	Sandy Loam	7.8	1.98	1.32	6.97	4.50	10.50	2.30	2.65	18.34

**Table 6:** Chemical analysis of water irrigation.

pH	EC (dS m^−1^)	Cations (meq L^−1^)				Anions (meq L^−1^)			SAR
		Ca^+2^	Mg^+2^	Na^+1^	K^+1^	HCO_3_^−1^	Cl^−1^	SO_4_^−2^	
8.11	0.92	4.5	1.14	3.5	0.15	2.12	2.43	3.22	1.52

## Data Availability

All data are presented within the article.
